# Ediacaran Marine Redox Heterogeneity and Early Animal Ecosystems

**DOI:** 10.1038/srep17097

**Published:** 2015-11-24

**Authors:** Chao Li, Noah J. Planavsky, Wei Shi, Zihu Zhang, Chuanming Zhou, Meng Cheng, Lidya G. Tarhan, Genming Luo, Shucheng Xie

**Affiliations:** 1 State Key Laboratory of Biogeology and Environmental Geology, China University of Geosciences, Wuhan 430074, China; 2 Department of Geology and Geophysics, Yale University, New Haven, CT 06520, USA; 3 LESP, Nanjing Institute of Geology and Palaeontology, Chinese Academy of Sciences, Nanjing 210008, China

## Abstract

Oxygenation has widely been viewed as a major factor driving the emergence and diversification of animals. However, links between early animal evolution and shifts in surface oxygen levels have largely been limited to extrapolation of paleoredox conditions reconstructed from unfossiliferous strata to settings in which contemporaneous fossils were preserved. Herein, we present a multi-proxy paleoredox study of late Ediacaran (ca. 560-551 Ma) shales hosting the Miaohe Konservat-Lagerstätte of South China and, for comparison, equivalent non-fossil-bearing shales at adjacent sections. For the fossiliferous strata at Miaohe there is geochemical evidence for anoxic conditions, but paleontological evidence for at least episodically oxic conditions. An oxygen-stressed environment is consistent with the low diversity and simple morphology of Miaohe Biota macrofossils. However, there is no evidence for euxinic (anoxic and sulphidic) conditions for the fossiliferous strata at Miaohe, in contrast to adjacent unfossiliferous sections. Our results indicate that Ediacaran marine redox chemistry was highly heterogeneous, even at the kilometre-scale. Therefore, our study provides direct—rather than inferred—evidence that anoxia played a role in shaping a landmark Ediacaran ecosystem. If the anoxic conditions characteristic of the studied sections were widespread in the late Neoproterozoic, environmental stress would have hindered the development of complex ecosystems.

The diversification of eukaryotes, which culminated in the emergence and diversification of animals in the late Neoproterozoic and earliest Cambrian, has long been viewed as the result of increasing oxygen levels in Earth’s atmosphere and, in turn, shallow and deep oceans^1^,^2^,^3^. The most basic aspects of this model are not contentious—animals have high metabolic oxygen demands relative to simple eukaryotes. However, there has been extensive debate, especially over the past decade, concerning whether there is compelling evidence that spatially widespread and temporally protracted anoxic marine conditions inhibited animal diversification and shaped early metazoan ecosystems^4^,^5^,^6^,^7^. Part of this debate stems from the fact that a limited amount of the redox work has been done in beds and deposits in which early animal ecosystems are preserved. Previous paleoredox work has instead focused predominantly on reconstructing basin- or global-scale paleoredox conditions^3^,^8^,^9^,^10^. Although this broader-scale work is, without question, essential to reconstructing Earth’s oxygenation, the links between biological and ecological complexity and marine oxygen levels have long been extrapolated, rather than directly demonstrated. Ideally, geochemical study of broad-scale redox evolution would be coupled with paleontological analyses of the same deposits—the fossil deposits that are shaping our view of Neoproterozoic biological evolution. However, linking detailed paleontological and paleoredox work can be problematic; this work is dependent upon a fortuitous confluence of the appropriate lithology for geochemical analyses and *in situ* fossil preservation.

Miaohe black shales in South China provide one locality where detailed paleontological and paleoredox work can be done. Shales in the uppermost Ediacaran Doushantuo Formation (Member IV), exposed at Miaohe in the Yangtze Gorges area (Hubei province; Fig. 1), contain unusually well-preserved carbonaceous compressions (termed the “Miaohe Biota”), many of which have been interpreted as multicellular eukaryotic algae (mainly green, red and brown algae)^11^ and putative metazoans, such as the putative cnidarian or stem-group ctenophore *Eoandromeda octobrachiata*^11^,^12^,^13^,^14^,^15^. The presence of holdfasts in many Miaohe fossils and their commonly dense but non-overlapping distribution on bedding planes and high fidelity of preservation suggest that these algae were benthic and were preserved *in situ*^11^. Well-developed benthic communities would have required at least periodically oxic bottom-water conditions concurrent with deposition of the host strata. In order to explore whether oxygen levels may have controlled the composition and complexity of the Miaohe Biota ecosystem, we conducted a multifaceted paleoredox study of the fossiliferous Miaohe section and a coevally deposited (Member IV of the Doushantuo Formation) non-fossiliferous section at Jiuqunao (ca. 2 km south of Miaohe; Fig. 1b). We used trace metal data, Fe speciation, and petrographic characterization of sedimentary pyrites to determine water-column redox state during deposition of both fossiliferous and unfossiliferous shales. This work, in conjunction with previous work^9^ at an additional neighbouring coeval section at Jiulongwan (ca. 30 km southeast of Miaohe; Fig. 1b) provides us with a unique opportunity to investigate the relationship between water-column chemistry and ecosystem complexity in the late Ediacaran.

## Results

### Samples

**Figure 1:**
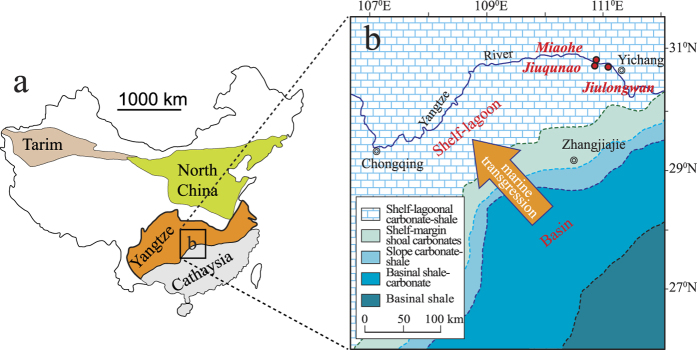
Geological context of studied sections. (a) The tectonic setting of South China in the Neoproterozoic. This map was made by Adobe Illustrator CS5. (b) Paleogeographic reconstruction of the Yangtze platform during deposition of the middle and upper Doushantuo Formation and the locations of the three sections included or discussed in this study. Modified from ref. 55 (DOI:10.1016/j.epsl.2007.07.009).

**Figure 2:**
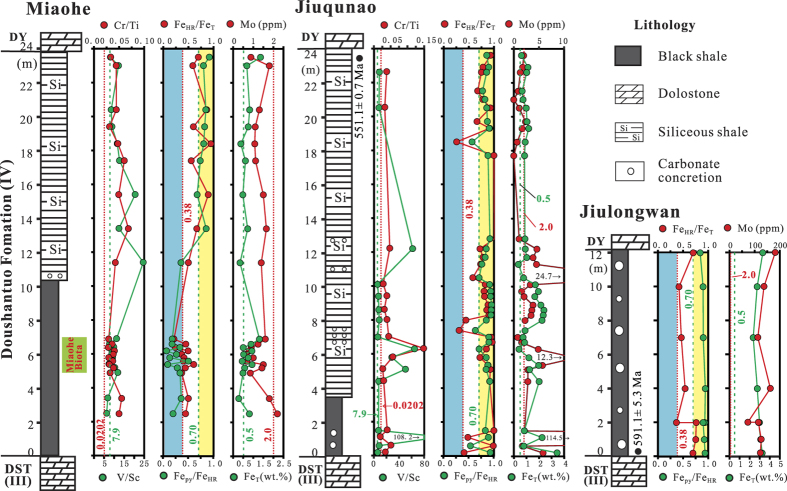
Composite stratigraphy with trace metal and iron speciation data that provide information on spatial and temporal variability in ocean chemistry concurrent with deposition of uppermost black shales and siliceous shales of the Doushantuo Formation. The interval in which the Miaohe Biota fossils are preserved is marked at the Miaohe section based on sample fossil examination and descriptions in ref. 11. The vertical dashed lines (from left to right) for each section indicates key values of 0.0202 (Cr/Ti), 7.9 (V/Sc), 0.38 (Fe_HR_/Fe_T_), 0.7 (Fe_py_/Fe_HR_), 0.5 (wt.%, Fe_T_) and 2 (ppm, Mo) used for geochemical analysis (see text). Data sources: (1) trace metal and Fe speciation data: Miaohe and Jiuqunao sections - this study and Jiulongwan section – ref. 9. (2) The chronological dating data marked at the Jiulongwan and Jiuqunao sections: 591.1 ± 5.3 Ma – ref. 21 and 551.1 ± 0.7 Ma – ref. 22.

Samples were collected from the Miaohe Biota-bearing black shales and overlying siliceous shales at the Miaohe section and from coevally deposited shales at the Jiuqunao section where Miaohe-type fossils are not found (Fig. 2). Large blocks (>200 g) were excavated and the freshest portions from these blocks (<50 g) were powdered for chemical analyses. We did not sample beds with macroscopic diagenetic pyrite nodules or bands. The sampled black shale intervals at both Miaohe and Jiuqunao, as well as coeval strata at Jiulongwan are characterised by planar and thinly laminated bedding and are interpreted to have been deposited during a basin-scale marine transgression on the Ediacaran Yangtze platform^16^,^17^ (Fig. 1b). Due to strong similarity in facies, coeval strata can be stratigraphically correlated between sections^18^ although uncertainty still remains^19^. The middle–upper Doushantuo Formation of the Yangtze Gorges area is interpreted to record deposition in a shelfal-lagoonal environment^20^ (Fig. 1b).

A Re–Os age of 591.1 ± 5.3 Ma for the black shales at the base of the shale-rich interval of Member IV^21^ (Jiulongwan section) and U-Pb age of 551.1 ± 0.7 Ma for a tuff bed from the top of the shale-rich interval^22^ (Jiuqunao section) were originally used to suggest that deposition of these shales may have lasted ~40 Ma. However, Kendall *et al.* (2015)^10^ suggested that the 591.1 ± 5.3 Ma Re-Os age may reflect post-depositional modification and proposed an alternate lower age bracket for Member IV of the Doushantuo Formation of ca. 560–551 Ma. This estimate is consistent with the occurrence of *Eoandromeda octobrachiata* not only at Miaohe, but also in the Ediacara Member of the Rawnsley Quartzite of South Australia, which, on the basis of taxonomic similarity to the well-dated Ediacara fossil assemblages of the White Sea region^23^, is likely also ca. 560–550 Ma^15^.

### Trace metals

The sedimentary enrichment of redox-sensitive metals such as chromium (Cr), vanadium (V) and molybdenum (Mo) provides a means to track local and, in certain cases, global redox conditions^24^,^25^,^26^. More specifically, it is possible to use the relative enrichment of different redox-sensitive metals to peg the presence of suboxic (water-column O_2_ < 5 μM), anoxic and euxinic (anoxic and sulphidic) conditions. In a suboxic to anoxic water column, Cr(VI) will be reduced and Cr(III) will be scavenged; in a fully anoxic water column V(V) will be reduced and V(IV) will be scavenged^24^. In a euxinic water column there will be reduction, sulfurization, and scavenging of Mo(VI) and, potentially, formation and scavenging of V(III)^24^. Therefore, Cr or V enrichments without strong Mo enrichments can be indicative of anoxic (but not euxinic) conditions while strong V and/or Mo enrichments are indicative of euxinic conditions^24^. This approach can be utilized despite uncertainties in the size of the dissolved marine metal reservoirs, given that Cr seems to be preferentially drawn down relative to Mo in anoxic Precambrian oceans^27^. The examined shales are comprised of sediments likely sourced from a terrane with a composition close to that of the average upper continental crust^28^, making it possible to use the relative abundance of detrital elements to estimate primary enrichment values for redox-sensitive metals^27^.

The examined sections contained significant Cr and V enrichments. We used t-test and one-sample t-tests to test for statistically significant differences from background crustal values (Cr/Ti = 0.0202, V/Sc = 7.9)^29^. Chromium and V were enriched relative to average upper continental crust values at both Miaohe and Jiuqunao: Cr/Ti = 0.0414 ± 0.0109 (SD) (*p* < 0.01) and V/Sc = 10.8 ± 4.3 (*p* < 0.01) for all Miaohe samples (n = 21); Cr/Ti = 0.0440 ± 0.0294 (*p* < 0.01) and V/Sc = 24.5 ± 30.5 (*p* < 0.05) for all Jiuqunao samples (n = 16) (Fig. 2)^29^. Low t-test *p*-values (*p* < 0.05) indicate that, these enrichments are statistically significant relative to shale composites^30^ and thus lend strong support to evidence for primary enrichments at both Miaohe and Jiuqunao. The examined South China sections are also enriched in Cr and V relative to the riverine samples that are the basis for the MUQ shale composite^31^. However, several individual samples lack Cr (n = 3 at Miaohe and n = 1 at Jiuqunao) and V (n = 4 at Miaohe and n = 9 at Jiuqunao) enrichments. We only observed significant Mo enrichments (>10 ppm) in Jiulongwan black shales and three samples from the Jiuqunao black shales (Fig. 2 and Supplementary Table 1), which suggest euxinic conditions concurrent with certain intervals of deposition at these localities. At the Miaohe section, Mo concentrations are all <3 ppm and are essentially crustal in value. These enrichments suggest persistent suboxic to anoxic but non-sulphidic conditions at Miaohe. The Miaohe Biota-bearing layers (~4.9–6.9 m) are characterised by lower Cr enrichments (Cr/Ti = 0.0342 ± 0.0046, n = 10) relative to enrichment values for the underlying black shales (<4 m; average Cr/Ti = 0.0527, n = 2, *p* < 0.01) and upper siliceous shales (~11–24 m; Cr/Ti = 0.0469 ± 0.0118, n = 9, *p* < 0.01) at Miaohe, as well as shales at the Jiuqunao section (Cr/Ti = 0.0440 ± 0.0294, n = 16, but *p* = 0.411). The low Cr enrichment values characteristic of the fossiliferous Miaohe strata are potentially consistent with only periodic anoxic or suboxic conditions, rather than persistent anoxia^24^. There is not notable positive correlation between chromium and nickel and there are not notable Ni enrichments in the examined sections (see supplementary Fig. 1). The absence of high concentrations of Ni, typically enriched in mafic rocks^30^, as well as the weak positive correlation between Ni and Cr provide further indication that elevated Cr/Ti ratios do not reflect a (mafic) detrital Cr source.

### Iron speciation

Iron speciation in clastic rocks is a well-established paleoredox proxy^8^,^9^,^32^. In order to further assess water-column chemistry concurrent with deposition at Miaohe, four highly reactive Fe (Fe_HR_) pools, including sulphide (Fe_py_), carbonate (Fe_carb_), oxide (Fe_ox_), magnetite (Fe_mag_) and total Fe (Fe_T_) were measured. The ratios of Fe_HR_/Fe_T_ and Fe_py_/Fe_HR_ can be used to peg anoxic conditions or close proximity to anoxic water masses (strong Fe_HR_ enrichments, Fe_HR_/Fe_T_ > 0.38) and to determine whether samples reflect euxinic conditions (strong Fe_py_ enrichments, Fe_py_/Fe_HR_ > 0.7). The Fe speciation proxy has been previously calibrated through several studies of modern and ancient marine sediments^33^,^34^.

Iron ratios for the black shales at Miaohe section, like the trace metal data, are consistent with predominantly anoxic conditions or close proximity to anoxic water masses (0.21 ≤ Fe_HR_/Fe_T_ ≤ 0.61 and 0.07 ≤ Fe_py_/Fe_HR_ ≤ 0.5). The low iron enrichments characteristic of these strata could be indicative of either oxic or anoxic conditions. The overlying, unfossiliferous siliceous shales are characterised by iron ratios (0.50 ≤ Fe_HR_/Fe_T_ ≤ 0.95 and 0. 74 ≤ Fe_py_/Fe_HR_ ≤ 0.92, with two exceptions at 11.4 m and 15.4 m; Fig. 2) indicative of dominantly euxinic settings, although the lack of Mo enrichments (Fig. 2) would seem not to support this. Similarly, iron speciation values suggest that both the equivalent shales at Jiuqunao section were generally deposited under euxinic conditions (0.41 ≤ Fe_HR_/Fe_T_ ≤ 1.00 and 0.70 ≤ Fe_py_/Fe_HR_ ≤ 0.94 with two exceptions at 7.4 m and 18.5 m; Fig. 2). Previous Fe speciation (0.37 ≤ Fe_HR_/Fe_T_ ≤ 0.76 and 0.84 ≤ Fe_py_/Fe_HR_ ≤ 0.94) and Mo work has indicated that coeval strata at Jiulongwan were also deposited under euxinic conditions^9^. However, for a subset of the Miaohe and Jiuqunao samples, redox characterisation from Fe speciation is ambiguous, given the low Fe_T_ (<0.5 wt.%) characteristic of these samples (see Supplementary Table 1). The iron speciation proxy was not calibrated with sediment characterised by low total Fe values. Although all efforts were made to collect only well-preserved rocks possible, some samples from the Miaohe black shales (e.g., MH-5 and MH-9; Supplementary Table 1) contain a significant proportion of Fe_ox_, potentially indicating late-stage weathering of pyrites. However, the preservation of very fine-grained pyrite framboids in the examined shales (see below) suggests that secondary oxidation was limited. Therefore, the influence of pyrite weathering on Fe ratios should also have been limited.

### Pyrite framboids

Framboidal pyrite can be formed either in anoxic sedimentary pore fluids or in an anoxic water column^35^. Pyrite framboids formed in the water column are, on average, smaller and less variable in size than early diagenetic framboids^35^,^36^. For example, Wilkin *et al.* (1996)^35^ showed that the mean diameter of pyrite framboids in modern euxinic sediments is 5.0 ± 1.7 (SD) μm, whereas it is 7.7 ± 4.1 μm in oxic or dysoxic sediments. In addition, the maximum framboid diameter (MFD) is usually less than 20 μm for euxinic sediments, whereas it always is > 20 μm for oxic sediments^36^.

In order to further test our redox interpretation for the Member IV Doushantuo shales we carried out petrographic assessment of several representative samples (Table 1) characterised by trace metal and Fe proxy data suggesting anoxic conditions. The pyrite framboids are randomly disseminated in the examined samples (see Supplementary Fig. 2), and the average diameter of measured framboids ranges from 5.5 μm to 7.5 μm (with a very small SD; 1.4 μm–2.3 μm). Moreover, the MFD for all samples is less than 12 μm (see Supplementary Fig. 2). All of these features are consistent with water-column, rather than pore-fluid precipitation of pyrite framboids, providing independent evidence for deposition of the uppermost shales of the Doushantuo Formation of the Yangtze Gorges region under anoxic water-column conditions.

### Redox summary

**Figure 3:**
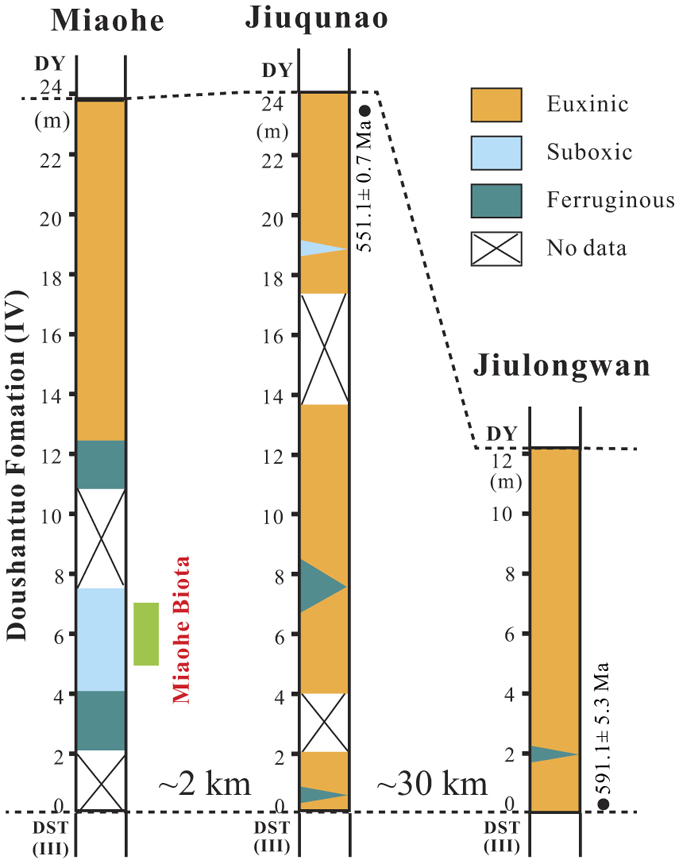
A graphic summary of water mass redox variations of the Doushantuo Formation (IV) among studied sections, as indicated by those integrated Fe and trace metal geochemical data and petrographic data of sedimentary pyrites presented in Fig. 2. See Fig. 2 caption for sources of chronological data.

In summary, the integrated trace metal, iron speciation and pyrite framboid size data suggest suboxic to anoxic but non-sulphidic bottom water conditions for the Miaohe-Biota-associated black shales, and more anoxic and increasingly sulphidic conditions for the overlying unfossiliferous siliceous shales and coevally deposited unfossiliferous shales at nearby sections (Fig. 3). These findings indicate that there was high spatial redox heterogeneity in the Ediacaran oceans, even on the kilometre scale.

## Discussion

Despite extensive study and exceptional preservation, fossil assemblages of the Miaohe Biota are characterised by relatively low diversity (~20 taxa) and are dominated by macroscopic algae^11^,^12^,^37^. *Eoandromeda octobrachiata*, one of the most convincing candidates from the Miaohe Biota for a fossil with metazoan or stem-metazoan affinity, has been interpreted to be a diploblastic-grade animal related to cnidarians or ctenophores^15^. In addition, the fossil *Cucullus* was recently interpreted to be a primitive demosponge^38^. However, in general, the putative metazoans, phylogenetic assignment of which, in many cases, remains inconclusive, constitute only a minor component, in terms of both species richness and relative abundance, of the Miaohe Biota ecosystem^11^,^12^,^37^. Furthermore, there is no firm evidence for complex bilaterians in the Miaohe Biota^11^. This is significant given that the complex bilaterians, including motile organisms, are typically major components of taphonomically similar Cambrian assemblages (e.g., Burgess Shale and Chengjiang Biota)^39^. The Miaohe Biota is a Konservat-Lagerstätte consisting largely of fossils preserved *in situ* and thus has the potential to capture a census population^11^,^40^. This suggests that the low-diversity assemblages and morphologically simple taxa preserved in the Miaohe Biota do not reflect a taphonomic bias but rather do indeed represent a low-diversity ecosystem characterised by a limited number of trophic levels.

The ecosystem structure of the Miaohe Biota would have been shaped by both evolutionary (e.g., the taxa that had evolved by the late Ediacaran) and environmental (e.g., ambient redox) factors. There is some uncertainty regarding the age of the Miaohe biota, but most likely it is between ca. 560 and 550 Ma (see above). Thus, it is possible, on the basis of coeval and older fossil deposits, to make general assumptions about what organisms could have been present and what ecological strategies would have been exploited at Miaohe. There is some evidence for benthic mobility, in the form of simple horizontal trace fossils from ca. 585–565 Ma strata^41^,^42^, although the age and trace fossil affinity of these structures have also been questioned. There is convincing trace fossil evidence for mobile bilaterians in the Ediacara Member of the Rawnsley Quartzite^43^ as well as other uppermost Ediacaran strata (e.g., Dengying Formation^44^). Regardless of direct (trace fossil) evidence for benthic animal mobility, there is molecular evidence that, by or even well before the mid-Ediacaran, some crown- and most stem-group metazoans had already appeared, including taxonomic groups (e.g., cnidarians) whose modern representatives are characterised by a mobile and have a predatory life mode^45^. Thus it is reasonable to suggest that, from an evolutionary perspective, the Miaohe could have supported a more complex ecosystem than is observed. Given the geochemical evidence for anoxia, the simple ecosystem structure at Miaohe can be linked to oxygen stress in the water column, although other environmental and ecological factors (e.g., water energy, temperature, nutrient availability, larval recruitment and settlement) must also be considered. Moreover, hypoxia in shallow marine environments, such as the Miaohe Biota-bearing strata are interpreted to record, is likely to be compounded by other environmental stresses (e.g., temperature variability, high-energy conditions). Water-column oxygen stress would have inhibited the development of energetically expensive lifestyles (e.g., mobility, predation) among benthic organisms and, at times, may have resulted in exclusion of all eukaryotes. Nearby sections with evidence for water-column euxinia are entirely lacking in macrofossils (Fig. 3), consistent with absence of or prohibitively low levels of oxygen and sulphide toxicity^9^. Had macroorganisms been present in the settings recorded by correlative strata, these sections likely would have captured a similar taphonomic window (i.e. these strata are characterised by a similar ‘taphonomic potential’) to the fossil assemblages at Miaohe, given strong evidence for euxinia and thus more reducing conditions which would have favoured organic carbon preservation at these sections. Therefore the lack of macrofossils in correlative strata in sections adjacent to Miaohe can be most parsimoniously interpreted as a true absence, and not a taphonomic artefact.

The Miaohe Biota provides a single snapshot of an Ediacaran community. However, the exceptional preservation characteristic of this deposit also provides unique insight into Ediacaran ecosystem-environmental interactions. Our integrated paleoredox data indicate suboxic to at least periodically anoxic but non-sulphidic water-column conditions concurrent with deposition of the Miaohe Biota-associated black shales. Further, our paleoredox work on nearby coeval non-fossiliferous black shales indicates that Ediacaran ocean chemistry was highly spatially heterogeneous, even at a scale of kilometres, and that both anoxia and euxinia were common components of this system (Fig. 3). The marine redox landscape will, foremost, be controlled by the extent (at both the local and basinal scales) of oxidant consumption through organic matter degradation. Patterns of ocean circulation and the extent of physical mixing within a water mass will also heavily influence marine redox state. Sulphate concentrations may drop to levels too low to sustain sulphate reduction, particularly in isolated basins with a strong freshwater input, but this scenario requires exceptionally low sulphate levels^9^,^46^,^47^. Lastly, high iron inputs (e.g., from hydrothermal sources and glacial sediments) can lead to locally ferruginous conditions^9^,^48^,^49^,^50^,^51^,^52^. Although it is difficult to gauge the exact role and relative balance of each of these factors in driving the observed redox heterogeneity, our results clearly demonstrate that these factors can indeed result in ecologically meaningful and highly spatially variable heterogeneity in redox conditions.

As the fossiliferous strata at Miaohe, although deposited under non-euxinic conditions, were deposited in very close proximity to strongly euxinic (and therefore toxic) environments, it is perhaps not surprising that the Miaohe ecosystems lived, died and were fossilized against a backdrop of dynamic redox variability and that the development, complexity and spatial distribution of Miaohe communities may have been strongly shaped by local water-column oxygen levels. If the low-oxygen conditions characteristic of Miaohe were common in late Ediacaran oceans, environmental stress would have suppressed the development of complex and evolutionarily stable ecosystems. This study, therefore, provides the first direct evidence for the model that the protracted radiation of animals was tied to Neoproterozoic redox evolution.

## Methods

### Iron speciation analysis

Four highly reactive Fe (Fe_HR_) pools (sulphide- Fe_py_, carbonate- Fe_carb_, oxide- Fe_ox_, and magnetite- Fe_mag_) and total Fe (Fe_T_) were measured. Content of Fe_py_ was calculated stoichiometrically from pyrite sulphur (FeS_2_) which was extracted by using CrCl_2_ reduction method and precipitated as Ag_2_S in silver nitrate traps^53^. One or two pure pyrite standards (Alfa Aesar) were extracted for each batch, generating a recovery of 93.3 ± 2.8% (mean ± SD, n = 9). Fe_carb_ Fe_ox_, and Fe_mag_ were extracted sequentially using the method described by Poulton and Canfield (2005)^54^ and measured using an atomic absorption spectrophotometer. At present, there are no internationally certificated standards for sequential extraction of Fe species. Two laboratory shale standards (CUG-2 and CUG-3), and that were repeatedly tested in the Lyons biogeochemistry Laboratory at University of California in Riverside (UCR), were used for quality control during the sequential extraction of all studied samples. Duplicate analyses of CUG-2 generate a Fe_carb_ value of 0.13 ± 0.02% (mean ± SD, n = 7, UCR value = 0.11%), a Fe_ox_ value of 0.21 ± 0.02% (n = 7, UCR value = 0.20%), and a Fe_mag_ value of 0.11 ± 0.01% (n = 7, UCR value = 0.10). Duplicate analyses of CUG-3 generate a Fe_carb_ value of 1.21 ± 0.14% (mean ± SD, n = 4, UCR value = 1.26%), a Fe_ox_ value of 0.21 ± 0.02% (n = 4, UCR value = 0.20%), and a Fe_mag_ value of 0.52 ± 0.10% (n = 4, UCR value = 0.64).

### Elemental and pyrite-framboid analyses

Concentration of total Fe was determined by X-ray fluorescence (XRF). Replicate analyses of Chinese National Reference Material GBW07107 (i.e., GSR-5, a shale standard, n = 3) and GBW07108 (i.e., GSR-6, a muddy dolostone standard, n = 3) were each reproducible within 0.1% (SD) for the XRF analyses. Concentrations of trace metals were measured by using an Optimass 9500 quadrupole inductively coupled plasma mass spectrometer following a standard multi-acid digestion (HNO_3_–HCl–HF). Analyses of USGS standard (BHVO-2), IRMM standard (BCR-2), GSR-5 and GSR-6 along with samples offered an analytical error of <6.8%, <3.3%, <9.0%, <5.9% and <19.7% for assays of Cr, Ti, V, Sc and Mo, respectively. A C-S analyzer (multi EA 4000) was used to measure total carbon (TC), total inorganic carbon (TIC) and total organic carbon (TOC = TC−TIC) through online combustion at 1350 °C and acidification with ~30–40% phosphoric acid, respectively. Analytical errors are <±0.2% for TC and TIC based on replicate analyses of Alpha Resources standards AR 4007 and AR 1034. Pyrite framboids were measured using a scanning electron microscope (Quanta 450 FEG) in backscattered electron mode.

### Statistics

We used one-sample t-tests in order to test for statistically significant differences from background crustal values (Cr/Ti = 0.0202, V/Sc = 7.9). We performed a Kolmogorov-Smirnov test to confirm that metal enrichments from the examined sections were normally distributed before performing a t-test. We also compared sections to the samples that make up the MUQ shale composite. Further, we used independent-sample t-tests in order to test for statistically significant differences from Cr/Ti values of the Miaohe Biota-bearing layers, which involve a Levene test for homogeneity of variances and a t-test. *P*-values of < 0.05 were assumed to be statistically significantly different. All *p*-values were calculated in SPSS software.

## Additional Information

**How to cite this article**: Li, C. *et al.* Ediacaran Marine Redox Heterogeneity and Early Animal Ecosystems. *Sci. Rep.* **5**, 17097; doi: 10.1038/srep17097 (2015).

## Supplementary Material

Supplementary Information

## Figures and Tables

**Table 1:** The size statistics of pyrite framboids in the uppermost shales of the Doushantuo Formation at the Miaohe and Jiuqunao sections.

Sample	Lithology*	Height (m)	Measurements	Mean framboid diameter (μm)	Maximum framboid diameter (μm)	Standard Deviation
Miaohe Section						
MH-22	SS	23	128	7.52	11.30	2.04
MH-18	SS	18.4	172	7.53	11.85	2.29
MH-6	BS	5.6	184	6.56	11.15	1.83
MH-3	BS	4.9	608	6.05	11.3	1.86
Jiuqunao Section						
JQN-21	SS	10.5	352	7.14	10.56	2.2
JQN-11	SS	7	224	7.32	10.59	1.92
JQN-3	BS	1.1	188	6.88	10.47	1.72
JQN-1	BS	0.2	180	5.53	9.18	1.40

*SS = siliceous shale; BS = black shale.
