# Supplementary material for: Ediacaran Marine Redox Heterogeneity and Early Animal Ecosystems
Source: Sci Rep. 2015 Nov 24;5:17097. doi: 10.1038/srep17097 (PMC4656985; doi:10.1038/srep17097)
Supplement: Supplementary Information [file srep17097-s1.pdf]

Supplementary Information

for

“Ediacaran Marine Redox Heterogeneity and Early Animal Ecosystems”

by

Chao Li, Noah J. Planavsky, Wei Shi, Zihu Zhang, Chuanming Zhou, Meng Cheng, Lidya G. Tarhan, Genming Luo, Shucheng Xie

Supplementary Figure.

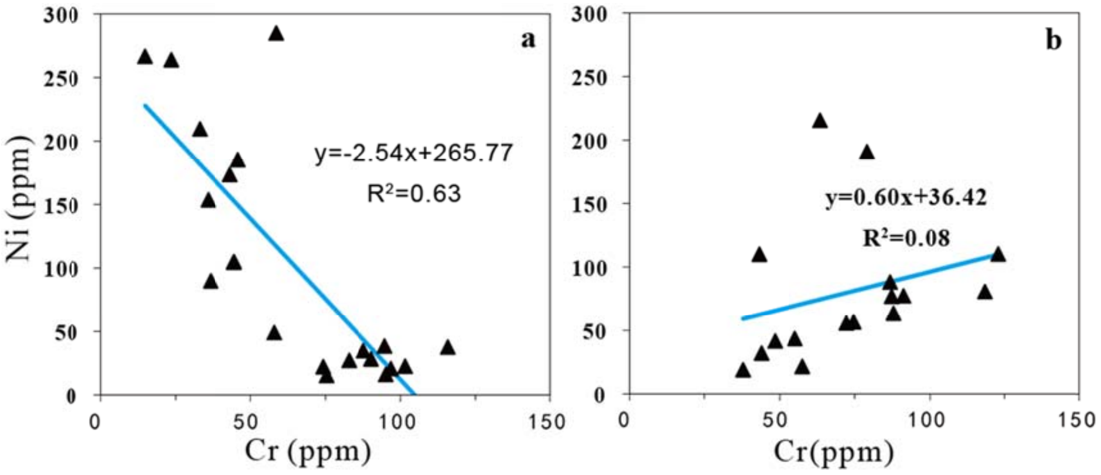

Supplementary Figure 1. Crossplots of Ni versus Cr for samples from Miaohe section (a) and from Jiuqunao section (b).

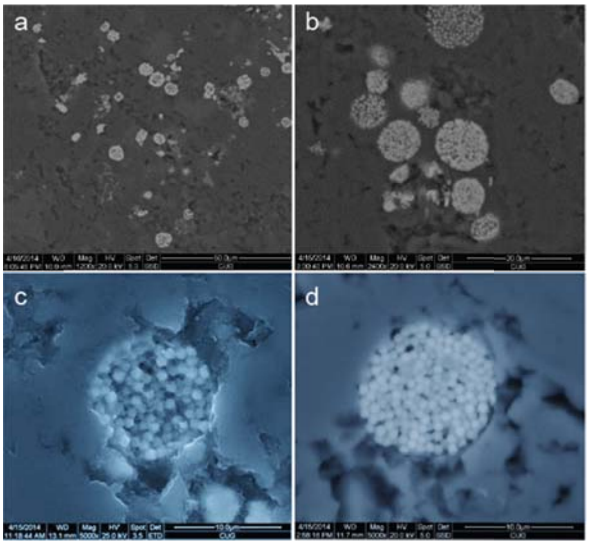

Supplementary Figure 2. Images of typical pyrite framboids in studied Doushantuo shales. (a-b) random distribution of the pyrite framboids in thin sections. (c-d) a close look of the framboids. The scale bars in A-D are 50μm, 20μm, 10μm and 10μm, respectively.

Supplementary Table 1

Summary of key geochemical data discussed in this study for the topmost shales of the Doushantuo Formation (Member IV) at the Miaohe, Jiuqunao and Jiulongwan sections

Sample	Height (m) [*]	Lithology [†]	TOC (wt.%)	Cr/Ti	V/Sc	Mo [§] (ppm)	Mo [#] (ppm)	Fe_{carb} (wt.%)	Fe_{ox} (wt.%)	Fe_{mag} (wt.%)	Fe_{py} (wt.%)	Fe_{HR} (wt.%)	Fe_T (wt.%)	Fe_{HR}/Fe_T	Fe_{py}/Fe_{HR}
Miaohe section															
MH-23	23.5	SS	0.3	0.0334	8.2	0.9	1.2	0.03	0.04	0.01	0.87	0.95	1.36	0.70	0.92
MH-22	23.0	SS	2.3	0.0443	12.1	1.8	2.1	0.05	0.02	0.01	0.32	0.40	0.68	0.59	0.80
MH-20	20.4	SS	1.8	0.0446	8.6	1.3	0.8	0.07	0.03	0.01	0.60	0.71	0.82	0.87	0.85
MH-19	19.4	SS	1.3	0.0311	8.9	1.1	1.6	0.04	0.03	0.01	0.37	0.45	0.75	0.60	0.82
MH-18	18.4	SS	2.1	0.0476	11.5	1.1	1.4	0.02	0.04	0.01	0.29	0.36	0.38	0.95	0.81
MH-17	17.4	SS	0.8	0.0604	12.7	1.1	2.5	0.05	0.03	0.01	0.25	0.34	0.61	0.56	0.74
MH-15	15.4	SS	0.3	0.0494	20.5	1.5	2.9	0.04	0.09	0.01	0.29	0.43	0.48	0.90	0.67
MH-14	13.4	SS	1.6	0.0685	12.4	1.7	1.3	0.04	0.02	0.01	0.41	0.48	0.72	0.67	0.85
MH-13	11.4	SS	0.5	0.0426	24.5	1.4	0.9	0.04	0.06	0.01	0.06	0.17	0.34	0.50	0.35
MH-12	6.9	BS	1.6	0.0293	11.2	1.6		0.03	0.17	0.03	0.05	0.28	1.31	0.21	0.18
MH-11	6.6	BS	2.0	0.0362	7.8	1.2	0.0	0.02	0.28	0.03	0.07	0.40	0.89	0.45	0.18
MH-10	6.4	BS	2.0	0.0286	9.8	0.9	0.0	0.01	0.06	0.01	0.04	0.12	0.50	0.24	0.33
MH-9	6.2	BS	2.1	0.0408	8.9	0.8	0.0	0.02	0.36	0.05	0.03	0.46	0.93	0.49	0.07
MH-8	6.0	BS	2.7	0.0384	8.8	0.6	0.0	0.02	0.11	0.01	0.05	0.19	0.52	0.37	0.26
MH-7	5.8	BS	2.3	0.0294	9.3	1.0	0.0	0.03	0.22	0.02	0.04	0.31	0.75	0.41	0.13
MH-6	5.6	BS	2.6	0.0375	9.2	0.7	0.0	0.02	0.06	0.01	0.09	0.18	0.45	0.40	0.50
MH-5	5.4	BS	2.6	0.0306	7.4	1.5	0.0	0.01	0.48	0.03	0.05	0.57	0.94	0.61	0.09
MH-4	5.2	BS	3.2	0.0389	10.3	1.4	0.0	0.01	0.15	0.02	0.07	0.25	0.57	0.44	0.28
MH-3	4.9	BS	2.2	0.0323	11.8	0.9	1.8	0.01	0.09	0.02	0.06	0.18	0.49	0.37	0.33
MH-2	3.4	BS	1.3	0.0553	6.8	1.8	2.1	0.02	0.06	0.01	0.05	0.14	0.28	0.50	0.36
MH-1	2.5	BS	0.9	0.0500	6.2	2.2	2.0	0.06	0.21	0.02	0.07	0.36	0.81	0.44	0.19
Jiuqunao section															
JQN-36	23.6	SS	0.0				2.0	0.05	0.02	0.01	0.48	0.56	0.60	0.93	0.86
JQN-35	22.9	SS	0.0				1.8	0.06	0.02	0.01	0.78	0.87	1.11	0.78	0.90
JQN-34	22.6	SS	0.0	0.0383	8.7	1.3	1.8	0.08	0.02	0.01	0.65	0.76	1.01	0.75	0.86
JQN-33	21.5	SS	0.9				0.9	0.07	0.02	0.01	0.34	0.44	0.65	0.68	0.77
JQN-32	21.0	SS	2.3				0.0	0.07	0.02	0.01	0.46	0.56	0.72	0.78	0.82
JQN-31	20.5	SS	1.9	0.0326	7.9	1.1	0.3	0.10	0.02	0.01	0.78	0.91	0.98	0.93	0.86
JQN-29	19.7	SS	0.8				2.4	0.06	0.02	0.01	0.60	0.69	1.04	0.66	0.87
JQN-28	19.3	SS	0.4				1.7	0.07	0.02	0.01	0.97	1.07	1.17	0.91	0.91
JQN-27	18.5	SS	0.6				0.7	0.07	0.02	0.01	0.13	0.23	0.89	0.26	0.57
JQN-26	17.7	SS	0.0				0.0	0.07	0.02	0.01	0.74	0.84	0.84	1.00	0.88
JQN-25	12.8	SS	1.0				1.1	0.04	0.02	0.01	0.81	0.88	0.87	1.00	0.92
JQN-24	12.2	SS	0.6	0.0473	61.5	4.6	4.2	0.07	0.02	0.01	0.55	0.65	0.90	0.72	0.85
JQN-23	11.7	SS	0.7				3.8	0.11	0.02	0.01	0.66	0.80	1.02	0.78	0.83
JQN-22	11.3	SS	0.5				4.5	0.04	0.02	0.00	0.19	0.25	0.34	0.74	0.76
JQN-21	10.5	SS	2.4				24.7	0.10	0.02	0.01	0.31	0.44	0.76	0.58	0.70
JQN-20	10.1	SS	0.9	0.0280	5.7	3.3	0.8	0.08	0.03	0.01	1.29	1.41	1.70	0.83	0.91
JQN-19	9.7	SS	0.9				1.6	0.07	0.02	0.01	1.46	1.56	1.94	0.80	0.94
JQN-18	9.4	SS	0.8	0.0388	7.1	2.1	3.1	0.06	0.02	0.01	1.25	1.34	1.65	0.81	0.93
JQN-17	8.9	SS	0.8				3.8	0.06	0.02	0.01	1.80	1.89	2.14	0.88	0.95
JQN-16	8.6	SS	1.4	0.0311	7.6	3.6	2.3	0.08	0.02	0.01	1.93	2.04	2.38	0.86	0.95
JQN-15	8.3	SS	1.9				3.4	0.09	0.03	0.01	1.97	2.10	2.33	0.90	0.94
JQN-14	8.0	SS	2.3	0.0391	8.4	2.2		0.10	0.04	0.01	0.84	0.99	2.23	0.44	0.85
JQN-12	7.4	SS	2.8					0.06	0.03	0.01	0.17	0.27	0.88	0.31	0.63
JQN-11	7.0	SS	1.7	0.0441	6.0	0.8	0.7	0.04	0.02	0.01	0.61	0.68	0.72	0.94	0.90
JQN-10	6.7	SS	2.1				1.6	0.05	0.02	0.01	0.73	0.81	0.84	0.96	0.90
JQN-9	6.3	SS	1.9	0.1484	64.4	4.9	3.8	0.06	0.03	0.01	0.24	0.34	0.40	0.85	0.71
JQN-8	5.8	SS	3.2	0.0566	28.2	12.3	7.7	0.06	0.06	0.01	0.72	0.85	1.18	0.72	0.85
JQN-7	5.35	SS	2.6				5.7	0.11	0.09	0.01	1.52	1.73	1.96	0.88	0.88
JQN-6	5.1	SS	2.4	0.0265	50.1	2.8	3.5	0.13	0.04	0.01	0.97	1.15	1.24	0.93	0.84
JQN-5	4.4	SS	2.1	0.0293	7.5	2.8	0.0	0.10	0.04	0.01	1.68	1.83	2.00	0.92	0.92
JQN-4	1.5	BS	0.9	0.0397	7.1	2.1	0.0	0.10	0.03	0.01	0.68	0.82	0.82	1.00	0.83
JQN-3	1.1	BS	6.8	0.0192	108.2	114.5	87.1	0.05	0.06	0.01	0.96	1.08	2.24	0.48	0.89

JQN-2	0.6	BS	2.2	0.0508	8.1	1.7	2.9	0.33	0.02	0.01	0.41	0.77	0.78	0.99	0.53
JQN-1	0.2	BS	5.2	0.0341	5.9	5.8	9.1	0.05	0.04	0.01	1.29	1.39	3.43	0.41	0.93
Jiulongwan Section [¶]															
HN-23	12.0	BS	15.1			180.7		0.20	0.12	0.04	1.92	2.29	3.26	0.70	0.84
HN-21	10.0	BS	5.2			136.0		0.07	0.02	0.02	1.02	1.13	2.70	0.42	0.90
HN-18	7.0	BS	6.4			111.4		0.06	0.03	0.01	0.97	1.07	2.30	0.47	0.90
HN-15	4.0	BS	5.0			161.3		0.05	0.01	0.02	1.38	1.47	2.71	0.54	0.94
HN-13	2.0	BS	4.8			71.8		0.05	0.01	0.01	1.01	1.08	2.93	0.37	0.93
HN-09	2.0	BS	4.6			113.9		0.09	0.11	0.02	1.95	2.16	2.84	0.76	0.90
HN-12	1.0	BS	5.1			125.1		0.10	0.07	0.01	2.12	2.30	3.05	0.75	0.92
HN-11	0.2	BS	2.0			121.7		0.07	0.06	0.01	2.09	2.23	3.20	0.70	0.94

* All heights are given relative to the base of the member IV of the Doushantuo Formation.

[†] SS = siliceous shale; BS = black shale.

[§] Mo concentration was measured by ICP-MS following a standard multi-acid digestion (HNO_3–HCl–HF).

[#] Mo concentration was measured by portable XRF fluorescence.

[¶] Data are compiled from Reference 1.

Reference

1. Li, C., *et al*. A Stratified Redox Model for the Ediacaran Ocean. *Science* **328**, 80–83 (2010).
